# Predictions and rewards affect decision-making but not subjective experience

**DOI:** 10.1073/pnas.2220749120

**Published:** 2023-10-25

**Authors:** Nicolás Sánchez-Fuenzalida, Simon van Gaal, Stephen M. Fleming, Julia M. Haaf, Johannes J. Fahrenfort

**Affiliations:** 1 Department of Psychology, University of Amsterdam, The Netherlands; 2 Amsterdam Brain & Cognition, University of Amsterdam, The Netherlands; 3 Wellcome Centre for Human Neuroimaging, Institute of Neurology, University College London, UK; 4 Department of Experimental Psychology, University College London, UK; 5 Max Planck Centre for Computational Psychiatry and Ageing Research, University College London, UK; 6 Department of Applied and Experimental Psychology, Vrije Universiteit Amsterdam, The Netherlands

**Keywords:** consciousness, perceptual decision-making, decision bias, rewards, predictions, visual illusion

## Abstract

To survive, organisms constantly make decisions to avoid danger and maximize rewards in information-rich environments. As a result, decisions about sensory input are not only driven by sensory information, but also by other factors, such as the expected rewards of a decision (known as the payoff matrix) or by information about temporal regularities in the environment (known as cognitive priors or predictions). However, it is unknown to what extent these different types of information affect subjective experience, or whether they merely result in non-perceptual response criterion shifts. To investigate this question, we used three carefully matched manipulations that typically result in behavioral shifts in decision criteria: a visual illusion (Müller-Lyer condition), a punishment scheme (payoff condition), and a change in the ratio of relevant stimuli (base rate condition). To gauge shifts in subjective experience, we introduce a novel task in which participants not only make decisions about what they have just seen, but are also asked to reproduce their experience of a target stimulus. Using Bayesian ordinal modeling, we show that each of these three manipulations affects decision criterion as intended, but that the visual illusion uniquely affects sensory experience as measured by reproduction. In a series of follow-up experiments, we use computational modeling to show that although the visual illusion results in a distinct drift-diffusion (DDM) parameter profile relative to non-sensory manipulations, reliance on DDM parameter estimates alone is not sufficient to ascertain whether a given manipulation is perceptual or non-perceptual.

Decision bias in perceptual decision making is a prevalent, well-known phenomenon (Green & Swets, 1966). But what is often not clear, even in simple perceptual tasks, is whether perceptual or non-perceptual processes are responsible for these biases. Sometimes the nature of the bias can be intuitively assumed to be perceptual, as in the Müller-Lyer illusion (Müller-Lyer, 1889), a phenomenon in which lines flanked by arrowheads appear as longer or shorter than they are on paper. However, there are many cases where the nature of the bias is not immediately clear, such as when manipulating the rewards contingent to a decision (payoff) or when altering the relative ratio of certain target stimuli (base rate).

Consider the following example: We put somebody in a dark room with a recurring faint light presented at threshold and ask her to detect the light to the best of her ability. After some time, we start imposing a large penalty for every missed light. Now the person reports the light more often, however her sensitivity is unaffected (as both the number of hits and the number of false alarms increases). Does she consciously see more lights after introducing the penalty? In this example, it is not immediately clear whether the observer is consciously perceiving a greater number of lights, or simply reporting the light more often without any change in their subjective experience due to a decisional-strategic change designed to maximize rewards.

When asked this question, two thirds of a group of attendees of the Association of Scientific Studies of Consciousness (ASSC) conference answered that the observer was not experiencing the light more often (Q2, Francken et al., 2022). Interestingly, there is currently little empirical data to convincingly argue either way. However, it is common practice in consciousness research to rely on subjects to accurately report what they experience. For example, to isolate a neural marker of conscious processing, or to establish whether different processes can function outside the scope of consciousness, trials on which participants indicate that they are conscious are typically contrasted with trials on which they indicate that they are not conscious (see Hannula et al., 2005 and Schmidt, 2015 for a review). However, without the ability to dissociate decisional from perceptual effects, it is unknown whether decision criterion shifts contaminate markers of conscious perception (Stein et al., 2016).

To separate criterion shifts from changes in performance, researchers have long relied on signal detection theory (SDT) (Green & Swets, 1966), as this framework quantifies bias and sensitivity independently. However, many researchers have come to equate criterion shifts with decisional effects and sensitivity shifts with perceptual effects. While it is true that measures such as *d’* and criterion differentially reflect sensitivity and bias, the latter can reflect both perceptual or non-perceptual response shifts (see Witt et al., 2015 for a detailed account). Accordingly, a number of studies have demonstrated that signal detection measures cannot be used as a principled method to distinguish between perceptual and non-perceptual biases (Morgan et al., 1990, 2013; Raslear, 1985; Witt et al., 2015).

To address this long-standing problem we combined a two-task experimental setup with Bayesian ordinal modeling (Haaf et al., 2018, 2021) to establish a framework for assessing whether decision criterion shifts are sensory or decisional in nature. We employed a standard decision task where observers discriminate between two categories, along with a novel reproduction task in which observers are asked to directly recreate their subjective experience in a controlled fashion. While the decision task is expected to be susceptible to changes in decision criteria (Morgan et al., 2013), we reasoned that the reproduction task should isolate perceptual effects. To pre-empt our findings, we show that the Müller-Lyer illusion biases both observers’ responses and subjective experience, whereas payoff and base rate manipulations bias only observer’s responses. In a series of follow-up experiments we show that applying computational modeling to reaction time data results in distinct drift diffusion parameter profiles between conditions, but is insufficient to identify in isolation whether a given decision criterion manipulation is perceptual or non-perceptual.

## Results

### Decision bias and length reproduction

**Figure 1:**
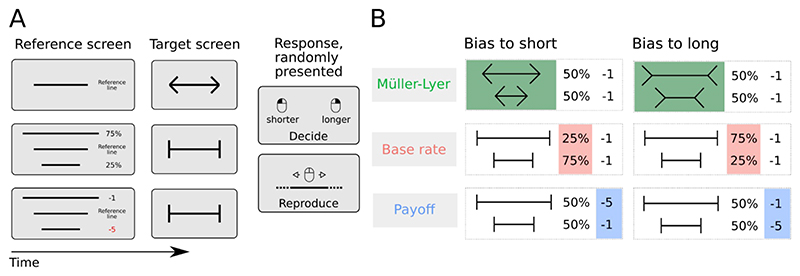
Experiment layout and bias manipulation summary. **A)** A typical sequence of trials, from here onward referred to as a mini-block, consisted of the presentation of a reference screen (until keypress) followed by five trials. Each trial consisted of a fixation period (500 ms), followed by a target screen (500 ms), followed by a second fixation period (600, 700 or 800 ms), finally followed by the prompt of one of the two tasks (the prompt was shown until an answer was registered). The Figure depicts an example of the reference screen and a target line for the Müller-Lyer biased to short (first row), base rate biased to long (middle row) and payoff condition biased to long (bottom row). The decision task consisted of a standard 2AFC task where observers discriminate between two categories (‘short’ and ‘long’). In the novel reproduction task observers were asked to directly recreate their subjective experience of the target line in a controlled fashion. **B)** Target lines presented in the Müller-Lyer condition were flanked by inward-pointing arrowheads when the bias direction was long and by outward-pointing arrowheads when the bias direction was short. In the base rate and payoff condition, vertical lines flanked the target lines. In the base rate condition there were three times more long lines than short lines when the bias direction was long and vice versa when the bias direction was short. In the Müller-Lyer and payoff conditions, there were an equal number of long and short trials. In the payoff condition participants lost 5 points for incorrectly answering long and 1 point for incorrectly answering short when the bias direction was short and vice versa when the bias direction was long.

We asked observers to categorize a series of target lines as being shorter or longer than a reference line (decision task), or alternatively, to reproduce their subjective experience of the target line length to the best of their ability (reproduction task) (see Figure 1A). Target lines were drawn from two distributions, one which contained lines that were longer and one which contained lines that were shorter than the reference line. The centers of the distributions were determined for each observer through a staircase procedure aimed at a 75% hit-rate performance (see methods for details). Every 5 trials, the reference line was repeated, to remind observers of its length. After viewing a target line, observers were given a prompt indicating whether they either had to make a 2AFC decision about the target line (shorter or longer than the reference line), or whether they had to directly reproduce the length of the target line. Crucially, observers did not know which task they would be performing while they viewed the target line, thus preventing specific task demands from affecting how the stimulus would be processed. Similarly, this also shielded our measures of decision bias and subjective experience from being directly affected by task context.

Further, in different blocks, observers were either biased towards the ‘short’ or towards the ‘long’ category. Three manipulations were used to bias observers: (1) target lines were either flanked by inward- or by outward-pointing arrowheads (Müller-Lyer illusion condition), (2) the ratio of target lines that were longer or shorter than the reference line was uneven, so one category was more prevalent (base rate condition), or (3) incorrect ‘long’ or ‘short’ decisions were differentially punished to bias responses towards the least costly option (payoff condition). See Figure 1B for a graphical depiction of the manipulations. We reasoned that if any of these bias manipulations would influence how the stimulus was perceived during the presentation of the target line, this effect should not only translate into a decision preference for the biased option, but also into a concomitantly biased line length reproduction.

**Figure 2:**
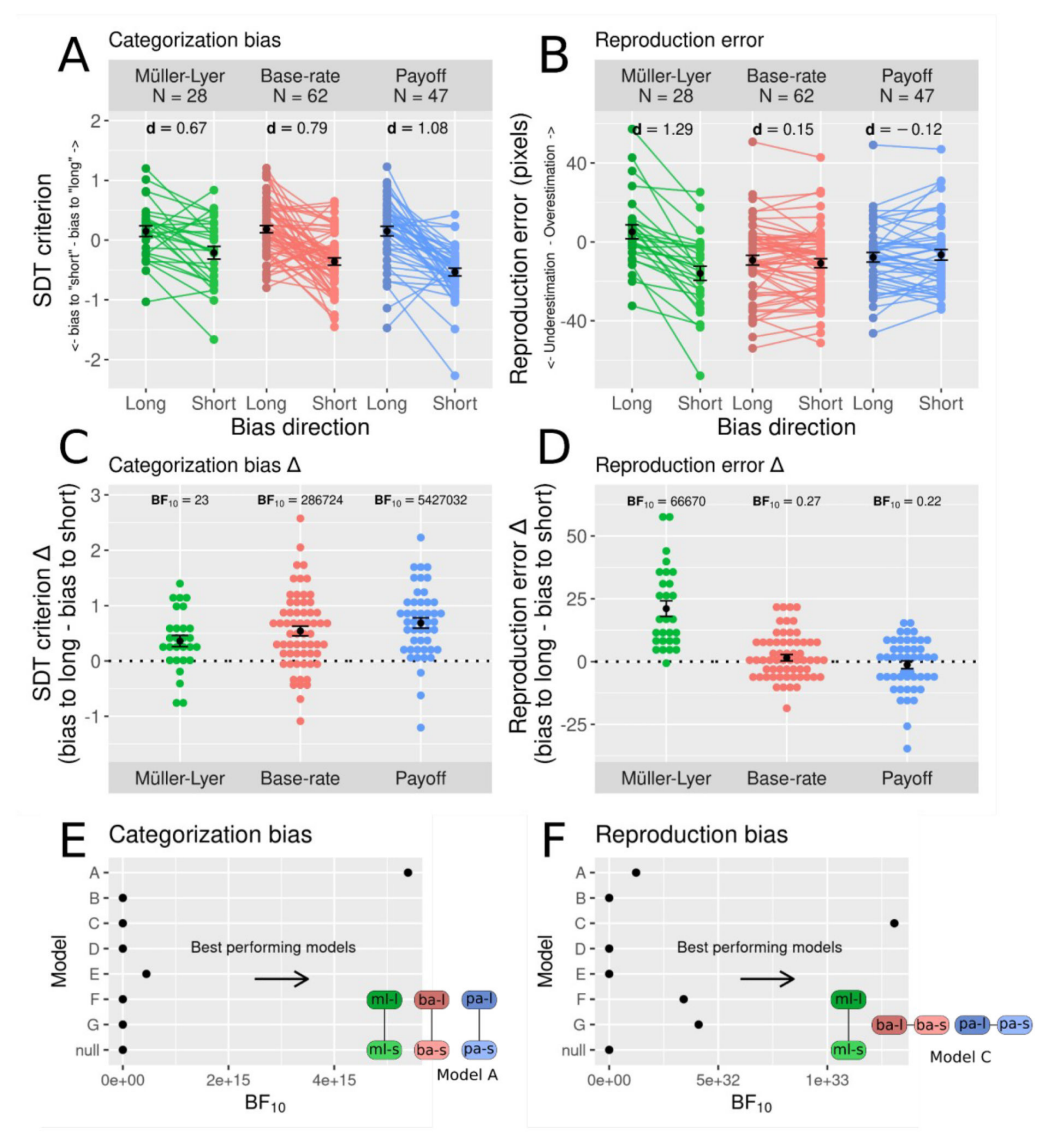
Main results reproduction experiment. **A) Categorization bias**. The SDT criterion value for each subject along with the group average for each bias source and bias direction condition. Higher values indicate a stronger bias towards answering ‘long’ while lower values indicate a stronger bias towards answering ‘short’. **B) Reproduction error**. The average reproduction error (*length reproduction - target length*) for each subject is displayed for each bias source and bias direction condition. Higher values indicate lines are reproduced as longer than the target line while lower values indicate lines are reproduced as shorter than the target line. **C and D)** The difference between bias direction long and short is displayed for the decision task and for the reproduction task. The higher the values in panels **C** and **D** the stronger the effect of each bias manipulation. **E and F)** Bayes factor values for each of the ordinal models tested. All models were compared against the null model. Higher values indicate a better performance of the model in comparison with the baseline model. For each task a graphical depiction of the winning model is included (model A in the decision task **(E)**, and model C in the reproduction task **(F)**). See supplementary Figure S3 for a graphical depiction of all models tested. All error bars indicate the standard error of the mean.

Across all conditions observers were able to distinguish between short and long lines (average SDT *d’* > 1; see Supplementary Fig. S1 for sensitivity data separated by condition). As a measure for bias in the decision task, we first calculated the mean bias (SDT criterion; see Methods) of each participant for each bias manipulation (Figure 2A) and computed the difference between the biases in the long- and short-bias conditions (Figure 2C). Overall, all manipulations resulted in large, positive effects, such that observers preferred the biased choice (‘short’ or ‘long’ depending on the bias direction), resulting in effect sizes (Cohen’s *d*) of 0.67 (Müller-Lyer), 0.79 (base-rate) and 1.08 (payoff). A simple paired Bayesian t-test with a default Cauchy prior of *√*2*÷*2 showed strong evidence for a bias direction difference in all bias manipulations (BF^10^ > 10 for all conditions). This default prior closely corresponds to medium effect sizes and is considered to be a good balance between non-informative and overly informative priors; see Wagenmakers et al. (2018) for further details.

Next, we wanted to determine whether these shifts in decisional bias also resulted in shifts in perception. As a measure of perceptual shifts, we calculated the mean reproduction error in the reproduction task (*reproduction length* - *target length*) of each participant for each bias manipulation (Figure 2B), and computed the reproduction error difference between long- and short-bias conditions (Figure 2D). While the Müller-Lyer condition showed a large effect (*d* = 1.29) reflecting the fact that the illusion led to the expected shifts in perception towards longer or shorter lines, the reproduction error magnitudes in the base rate and payoff conditions were nearly identical for the corresponding long- and short-bias conditions (*d* = 0.15 and *d* = -0.12). A simple paired Bayesian t-test with a default Cauchy prior of *√* 2*÷* 2 revealed extreme evidence for an effect in the Müller-Lyer condition (BF^10^ = 66670), and substantial evidence for a null effect in the base rate and payoff conditions (BF^10^ = 0.27 and BF^10^ = 0.22, respectively). To further check the validity of observers’ responses in the reproduction task we tested whether length reproductions correlated with the presented target line lengths. There was extreme evidence (BF^10^ > 100) for a large positive association between the length of the targets and observers’ length reproductions (*rho* = ~0.71, see Supplementary Fig. S2 for the exact Bayes factor and correlation coefficient values).

Although the effects we described above are consistent with a perceptual effect of the Müller-Lyer illusion and non-perceptual effects of the payoff and base rate manipulations, we devised a series of alternative models to account for other scenarios. To do this, we adopted a Bayesian model comparison framework to test for ordinal-constrained models (see Haaf et al. (2018) for an in-depth explanation of the method and Haaf et al. (2021) for a practical application). This statistical framework allows one to translate concrete, ordinal constellations of effects into statistical models that can be compared directly by computing their relative likelihood (see Analysis - Bayesian model comparison).

The outcome of these analyses confirmed our key results (see Figure 2E and F). In the decision task, the best performing model (A) was consistent with bias effects across all conditions (BF_A-over-null_ = 5.4e+15, Figure 2E), while in the reproduction task the best performing model (C) was one in which the Müller-Lyer led to a perceptual shift, while the base-rate and payoff had null effects (BF_C-over-null_ = 1.3e+33, Figure 2F; see Supplementary fig. S3 top panel for a graphical depiction of all models). Taken together, these data suggest that the Müller-Lyer illusion biases both decisions (categorization responses) as well as sensory experience (length reproductions), whereas the base rate and payoff manipulations bias responses without affecting subjective experience.

Could the apparent null effects of payoff and base-rate manipulations on reproduction actually reflect weak effects that are too small to be detected? Aside from the fact that the Bayesian approach generates explicit evidence for the null, it is unlikely that the null effects for payoff and base-rate during reproduction (Figure 2D) were caused by a lack of power to detect an effect, because these manipulations had the largest effect sizes in the decision task when compared to the Müller-Lyer (Figure 2C). To characterize such ordinal relationships between effects, we added a second set of models (see Supplementary fig. S3 bottom panel for a graphical depiction of these models) that further constrained the ordinal relationship across conditions and therefore tested the interaction of effect sizes across manipulations. In this second comparison, the best model (GG) indicated that the size of the effects in the payoff and base rate conditions were equal, and both were bigger than the Müller-Lyer effect in the decision task (BF_GG-over-A_ = 15.4; see Supplementary fig. S4 for the Bayes factor values of all models tested), effectively ruling out the possibility that the lack of an effect in the reproduction task for the payoff and base rate conditions is due to a weaker impact of the manipulation in these conditions. When testing the extended set of models in the reproduction task, the best performing model was still model C, in which only the Müller-Lyer condition influenced subjective experience (BF_C-over-A_ = 11), whereas the payoff and base-rate conditions exhibited null effects.

Another potential influence on our results might be how biases develop over time. In the Müller-Lyer manipulation, no previous experience is required for the illusion to have an effect. Conversely, the payoff and base-rate manipulations require some degree of learning to incorporate the contingencies and statistical regularities into the observer’s decision strategies. To minimize the influence of such learning effects during the task, we made the base-rate and payoff contingencies explicit to the observers during the practice session, and tested whether they understood these contingencies prior to the onset of the experiment (see General Procedure in Material and Methods). To further check that differences in learning could not account for our results, we evenly divided the experiment into three blocks to assess whether decision and reproduction results remained stable over time (experimental block) throughout the experiment. Models that only included bias direction as a factor were substantially more likely than models that included an interaction term between bias direction and experimental block. This was true both for the detection and for the reproduction experiment, and across all bias manipulations (see Supplementary fig. S5 for the decision and reproduction results by block and for a more detailed description of the analysis). Overall, these analyses suggest that learning during the experimental task played no differential role that could explain the differences between reproduction in the base rate and payoff conditions as compared to the Müller-Lyer condition.

## Computational modeling

Having established the non-perceptual character of the base rate and payoff manipulations, we employed three datasets using the same paradigm to determine whether computational modeling can be used to distinguish between perceptual and non-perceptual biases (as established in the reproduction experiment), but relying only on reaction times and choices measured in the decision task. To do this, we used drift diffusion models (DDMs) along with a very similar experimental design to the one previously presented, but without interleaving the tasks after stimulus offset. As before, observers were presented with a reference line, after which they had to categorize a series of lines as being shorter or longer than the reference line. In these experiments however, observers only had to perform the length categorization task on every trial, without having to wait for the task prompt. We opted for this setup as the delay between stimulus onset and task prompt in the initial set of experiments effectively erased the signatures of the reaction time profiles needed for drift diffusion modeling. Again, we used the same three bias manipulations: the Müller-Lyer illusion, a base rate and a payoff manipulation to bias participants towards answering ‘short’ or ‘long’ more often (see Methods).

**Figure 3:**
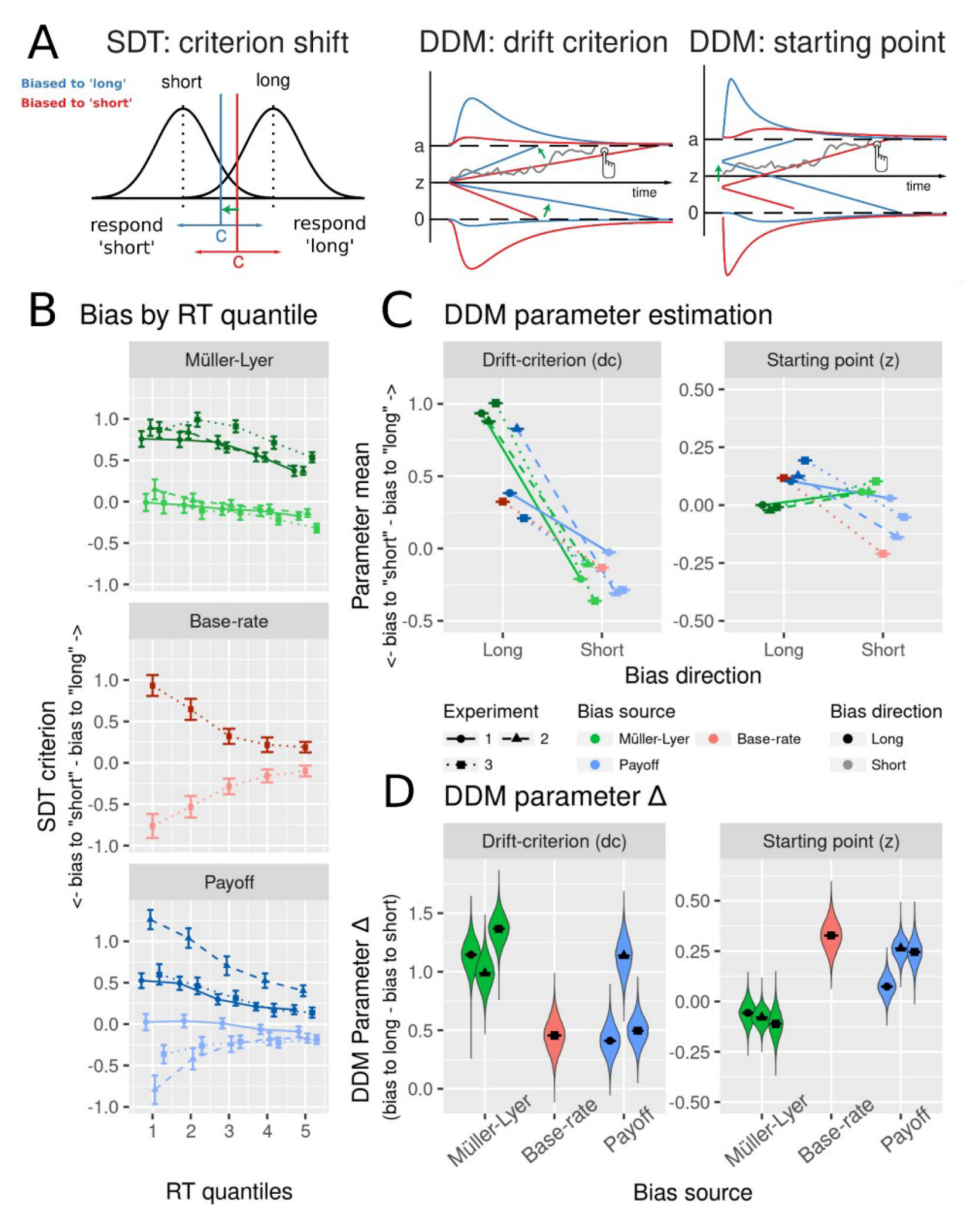
Theoretical accounts of decision bias and DDM results. **A, left panel)** SDT: criterion shift. Two distributions represent the strength of the shorter- and longer-line stimuli respectively. The decision threshold or criterion, determines whether a given stimulus is classified as being “short” or “long”. When the decision criterion shifts away from the midpoint between distributions, a greater number of stimuli is categorized as “short” (blue vertical line) or “long” (red vertical line). **A, middle panel)** DDM: drift criterion. When the evidence accumulation process has a non-zero drift criterion the evidence accumulates faster towards the biased-choice boundary. Red and blue lines depict two evidence accumulation processes with asymmetrical evidence drift-rate, so that the accumulation towards one boundary is faster. **A, right panel)** DDM: biased starting point. In drift diffusion models (DDMs) when the starting point (z) of the accumulation process shifts away from the midpoint between boundaries, less evidence is needed to reach the biased-choice decision boundary. Red and blue lines represent two evidence accumulation processes that started closer to one of the possible decision boundaries. **B)** Bias (SDT criterion) binned in RT quintiles (quintiles were calculated per subject and condition and then averaged across subjects). Positive values represent a bias towards reporting ‘long’ lines while lower values a bias towards reporting ‘short’ lines. **C)** Group-level posterior probability density of drift criterion and starting point parameters separated by bias condition and dataset. **D)** Bias strength is calculated as the difference between biased-to-short and biased-to-long parameter estimation for each condition and separately for starting point and drift criterion. Positive values indicate shifts in line with the expected decision bias (more stimuli classified as “long” when the bias direction is “long” and viceversa when the bias direction is “short”). Panel A is a modified version of Figure 1 in Kloosterman et al (2019).

DDMs assume a decision is made when noisy evidence accumulates from a starting point towards one of two response boundaries (Ratcliff, 1978). Reaction time (RT) distributions and choices can be used to model this evidence accumulation process. Unlike standard signal detection theory which has only a single parameter to quantify bias (Figure 3A, left panel), the drift diffusion framework contains two potential parameters to model bias: either the starting point shifts closer to one boundary (Figure 3A, right panel), resulting in less evidence needed to make a decision, or the drift criterion parameter biases the drift rate (Figure 3A, middle panel), so that the evidence accumulation process drifts faster towards the biased-choice boundary (Ratcliff, 1985; Ratcliff et al., 1999; Ratcliff & McKoon, 2008). Although the literature is not entirely consistent, recent research applying DDMs has predominantly associated base rate and payoff manipulations with starting point shifts, while perceptual-like manipulations have been associated more strongly with drift criterion shifts (Dekel & Sagi, 2020; Kloosterman et al., 2019; Leite & Ratcliff, 2011; Summerfield & de Lange, 2014; C. White & Poldrack, 2014; Yang et al., 2014). Although the existence of such parameter shifts seems reasonably well-established, the perceptual or non-perceptual nature of the manipulations studied have merely been implied rather than shown (as we have done here). Below, we make a direct link between the results from our reproduction task and a drift diffusion model fit to substantiate the inference that starting point shifts reflect non-perceptual, or strategic, decision biases on the one hand, and drift criterion effects reflect perceptual biases on the other hand.

Theoretically, the drift diffusion model predicts different reaction time (RT) distributions depending on whether the starting point or drift criterion is biased (Ratcliff, 1985, 2002). Indeed, simulated data with either biased starting point or drift criterion parameter values have shown that when the starting point shifts towards one boundary, decision bias is stronger for fast rather than slow responses, while shifting the drift criterion expresses itself as a bias in both fast and slow responses (White & Poldrack, 2014). Following White and Poldrack (2014), we binned the reaction times of each bias manipulation into quintiles and calculated a measure of bias within each bin (SDT criterion; see Figure 3B, see also Supplementary fig. S5 for the criterion and sensitivity data plotted by condition) to assess in a model-free manner whether we find such effects in our own data. Both the base rate and payoff condition show a very strong bias in the fastest responses that decreases as the RTs become slower, consistent with a starting point effect. On the other hand, the bias effect in the Müller-Lyer condition seems to stay roughly the same throughout the entire RT distribution, consistent with an effect on drift criterion.

To assess whether changes in drift criterion or starting point parameters were better able to capture the effect of the bias manipulations we fit a DDM model to each dataset where both starting point and drift criterion were allowed to vary as a function of bias source (Müller-Lyer, payoff and base rate) and bias direction conditions. Although we aimed to keep the other parameters (non-decision time, boundary separation and drift rate) equal across bias-source manipulations by keeping the experimental designs as similar as possible, in practice, it is impossible to control such parameters experimentally. Therefore, we allowed boundary separation, non-decision time and drift-rate to vary across bias sources in all models, but fixed them within bias sources (i.e. fixing them across different bias directions). In this setup, although one drift-rate is estimated for both bias directions within each bias source, the biased evidence accumulation rate for a given condition, or drift-bias (Ratcliff & McKoon, 2008), is the combination of the drift-criterion (dependent on the bias direction manipulation) and the stimulus-dependent drift-rate. We also included across-trial drift rate variability at the group level, as this additional parameter was found to improve fits to empirical RT data (Ratcliff & McKoon, 2008) but also because the length of long and short lines was drawn from a normally shaped distribution titrated for each subject, which resulted in small difficulty variations across trials of the same category within subjects. In order to ensure the model properly described the empirical data, we simulated new data (500 samples) for each subject using the fitted parameters of the model. We binned this simulated data in RT quantiles and plotted it to show that the model properly describes the decision bias patterns and RT profiles in the empirical data (see Supplementary fig. S6, see also S7 and S8).

Figure 3C shows the mean plus standard error around the mean of the full marginal posterior distribution of the drift criterion and starting point parameters for each of the conditions and for each dataset (see Supplementary fig. S9 for the full marginal posterior distribution of all parameters). The x-axis denotes bias direction (bias to long vs bias to short), so that the difference between parameter estimations across the x-axis reflect the efficacy of a given manipulation in affecting starting point or drift criterion. To quantify the effect of each bias manipulation more clearly, we calculated the difference between the biased to ‘long’ and biased to ‘short’ conditions for each bias manipulation on each experiment (see Figure 3D). These results clearly show that although both perceptual (Müller-Lyer) and non-perceptual manipulations (base-rate and payoff) affect drift criterion, perceptual and non-perceptual manipulations result in distinct parameter profiles relative to each other. For example, the effect on drift criterion is generally much larger in the Müller-Lyer condition than in the base-rate and payoff conditions (with the exception of experiment 2, where the payoff effect is larger; see Figure 3C and D, left panel). Furthermore, unlike in all the other conditions, the Müller-Lyer manipulation shows little to no effect in the starting point parameter, and if anything is in the opposite direction compared to the other conditions (see Figure 3C and D, right panel). Although there seems to be a small effect opposite to the direction of the manipulation (meaning that fast biased responses were directed to the non-biased choice, i.e. towards answering ‘short’ when the bias direction is ‘long’), it is worth mentioning that the fitted model does not perfectly recover fast responses of the choice opposite to the bias direction (see Supplementary fig. S8, Müller-Lyer biased to long panels), making it hard to confidently conclude that there is indeed an opposite effect in starting point in the Müller-Lyer condition.

Thus, when comparing multiple manipulations that use the same task (as we do here), there are multiple hints that the drift criterion parameter is more affected by perceptual manipulations than non-perceptual manipulations, while for the starting point parameter the opposite is true. However, when obtaining these parameter estimates from a single condition, it would be hard to conclude with certainty whether that condition contains a perceptual or a non-perceptual manipulation. The reason for this is two-fold: (1) we observe that the manipulations are not guaranteed to uniquely affect starting point or drift criterion in isolation, without also affecting the other parameter and (2) the parameter profile (the relative contribution of drift criterion and starting point) can only be assessed when compared against other manipulations, that is, parameter estimates from a single condition do not provide conclusive information.

One of the strengths in the current design is that the three bias manipulations were executed in an identical task setting. This allowed us to identify distinct parameter profiles when compared against each other (stronger/weaker drift criterion effects, or even opposite starting point effects depending on the bias manipulation), but it does not allow one to identify whether any given criterion manipulation is perceptual or non-perceptual without assessing the relative contribution of each parameter in relation to other bias manipulations.

**Figure 4:**
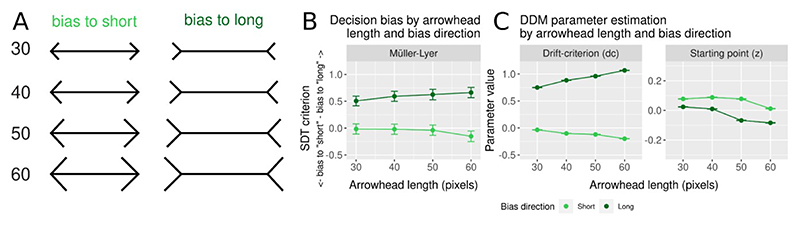
Müller-Lyer arrowhead length behavioral and DDM results. **A)** For each bias direction four different arrowhead lengths were tested (30, 40, 50 and 60 pixels; Figure not to scale). **B)** Average SDT criterion for each arrowhead length and for each bias direction. Higher values indicate a stronger bias towards answering ‘long’ while lower values indicate a stronger bias towards answering ‘short’. **C)** Group-level posterior probability density of drift criterion (left panel) and starting point (right panel) parameters separated by bias direction.

To further quantify the relation between the Müller-Lyer illusion and the drift criterion and starting point parameter, we varied the length of the arrow heads of the Müller-Lyer in experiment 2 (see Figure 4A). Increasing the length of the arrow heads is known to increase the strength of the illusion (Restle & Decker, 1977). We calculated the decision bias for each bias direction and arrowhead length, and indeed, decision bias further deviated from zero as the arrowhead length increased (Figure 4B). To assess whether the different arrowhead lengths had an effect on the RT profiles of the responses we again tested a model where starting point and drift criterion were allowed to vary with the bias direction of the manipulation, but also with the length of the arrowheads. Given the strong Müller-Lyer effect on drift criterion observed across all experiments, we expected the drift criterion to further deviate from zero as the length of the arrowheads increased.

Again we simulated data using the fitted parameter values of the model and plotted the predicted data to show that the model correctly describes the empirical data (Supplementary fig. S10, see also fig. S11). Figure 4C shows the drift criterion (left panel) and starting point (right panel) parameter estimation for each of the four arrowhead lengths tested. On the one hand, the drift criterion resembles the SDT criterion effect, as it shifts away from zero, either positively or negatively, depending on the bias direction. On the other hand, although the bias direction effect on the starting point parameter is inverted, it still shows a general shift downwards as the arrowhead length is increased. Again, as in the main DDM results, the effect of increasing the length of the Müller-Lyer arrowheads seems to load preferentially on drift criterion, however, there seems to be a more general effect of the arrowhead length, irrespective of the bias direction, that also loads on the starting point parameter. As before, the fitted model does not recover the fastest responses accurately when the selected choice is the opposite of the bias direction (see Supplementary fig. S11, Müller-Lyer biased to long panels), making it hard to interpret the inverted bias direction effect on the starting point parameter.

Taken together, these results show that, although drift diffusion modeling is able to distinguish between parameter profiles that underpin perceptual and non-perceptual manipulations, it can only do so relative to other manipulations. Importantly, unlike our novel reproduction task, these qualitative patterns do not allow DDMs to be used as a principled method to distinguish between perceptual and non-perceptual manipulations.

## Discussion

The goal of this study was to establish to what extent different types of information influence perceptual experience. To accomplish this, we evaluated the perceptual or non-perceptual nature of three well-known decision bias manipulations: the Müller-Lyer illusion, a base rate manipulation and a payoff manipulation. To assay perceptual experience, we designed a task where observers were either asked to categorize a series of target lines as being shorter or longer than a reference or to directly reproduce the length of each target line. To our knowledge, ours is the first task that is able to unequivocally distinguish between perceptual and non-perceptual decision criterion shifts. We first showed that the Müller-Lyer illusion biases both decisions as well as perceptual experience as measured in the reproduction task. In contrast, the base rate and payoff manipulations selectively biased decisions without affecting subjective experience. We then used computational modeling to show that perceptual and non-perceptual manipulations result in distinct DDM parameter profiles. However, because these profiles only allow one to make relative assessments, one cannot use DDM parameters in isolation to determine whether a task induces perceptual or non-perceptual changes in decision-making.

### Reproduction, but not discrimination, can distinguish between perceptual and non-perceptual biases

As expected, all bias manipulations resulted in the choice consistent with the bias direction being reported more often, and therefore in criterion shifts in the decision task. Regardless of the nature of the manipulation (perceptual/non-perceptual), the decision task was highly susceptible to bias. This is in line with previous research showing that 2AFC tasks are not only prone to bias but also incapable of distinguishing between perceptual and non-perceptual biases (Jogan & Stocker, 2014; Morgan et al., 2013) even when using SDT (Witt et al., 2015). A number of articles have tried to tackle this problem by using neuroimaging (for example, Balestrieri & Busch, 2021; Fleming et al., 2010; Ho & Schwarzkopf, 2022; Summerfield & Koechlin, 2010; see also de Lange et al., 2018) physiological measures (for example Pereira et al., 2021; A. White et al., 2022) and behavioral setups (for example, Gallagher et al., 2019; Jogan & Stocker, 2014; Linares et al., 2019; Morgan et al., 1990) that varied in both the complexity of their implementation but also in their degree of success. Here we showed that a controlled reproduction task provides a straightforward experimental approach that selectively captures the effect of perceptual manipulations on decision-making (as exemplified by the Müller-Lyer illusion), while showing no effect for non-perceptual manipulations exemplified by the payoff and base rate manipulations.

Although we kept the experimental design as similar as possible across conditions, it is worth considering two aspects of our experimental design that were consistently different in the Müller-Lyer condition as opposed to the payoff and base rate manipulations and the rationale behind them. Firstly, in the payoff and base rate condition, information about stimulus-response contingencies and base rate distribution were given during the reference screen, while in the Müller-Lyer condition, the arrowheads were placed around the target lines, instead of the reference line. However, it is important to realize that participants actually applied the information about contingencies in payoff and base rate to target lines, just as in the Müller-Lyer. For example, there are more long target lines when the base rate condition is biased to long. Similarly, in the payoff condition, the information given on the reference screen refers to how the length of the target line should be evaluated throughout the experiment, not how the reference line itself should be evaluated. Indeed, the payoff and base rate information conveyed on the reference screen did not change throughout a given condition and as such acted more as reminder rather than as something participants had to actively monitor each time the reference line was presented. Secondly, in the payoff and base rate condition we provided feedback separately for incorrect short and long responses in the decision task, while in the Müller-Lyer condition we only provided the total number of incorrect decision responses. In both cases the feedback information was intended to boost the efficacy of the manipulations. However, in the payoff and base rate condition response-specific feedback was necessary to keep participants aware of the stimulus-response contingencies, so that they would maintain a bias that optimized their rewards at a block level, while in the Müller-Lyer condition giving such response-specific feedback would have allowed them to correct their biased percept, which is why in that condition we only provided general feedback about the total number of incorrected decision responses. The feedback for the reproduction task was identical across all conditions (see Materials and Methods).

### Drift diffusion modeling shows different parameter profiles for perceptual versus non-perceptual bias manipulations

To answer whether our findings of selective perceptual biases were associated with unique behavioral profiles we explored the reaction time signatures of each bias manipulation using drift diffusion modeling. Across multiple datasets, we showed that although both perceptual and non-perceptual manipulations loaded on drift criterion, each bias type could also be associated with distinct parameter profiles when conditions were directly compared against each other.

It has been proposed that an optimal observer would have to adjust their evidence accumulation starting point, rather than drift criterion, when facing asymmetrical stimulus prevalence as well as reward scheme manipulations (Bogacz et al., 2006). While some empirical research has shown this to be the case (Leite & Ratcliff, 2011; Simen et al., 2009; White & Poldrack, 2014), others, more in line with our results, have found both starting point and drift criterion shifts in base rate (van Ravenzwaaij et al., 2012) and in payoff manipulations (Leite & Ratcliff, 2011). On the other hand, the Müller-Lyer illusion has been associated with drift rate effects (Schwarz & Reike, 2020) with similar findings for manipulations aimed at affecting perception such as when manipulating the length of a reference line (White & Poldrack, 2014). It is worth noting that these apparent mixed results are not limited to payoff and base rate manipulations. More recent studies have also found drift-criterion and starting point effects to result from prior information (Desai & Krajbich, 2022) and motivated reasoning (Gesiarz et al., 2019). The extent to which these manipulations are affecting the way stimuli is being perceived is hard to tell, but the mixed results in previous research may be caused by small differences in experimental designs not directly related to the manipulations of interest. It has been argued that the optimality of adjusting starting point exclusively, and/or in tandem with drift criterion, may depend on whether there is across-trial difficulty variation and whether the decision process is speeded, among other factors (see for example Hanks et al., 2011). For example, one could argue that a long enough evaluation should allow a decision maker to determine the true identity of the stimuli, rendering contextual biases irrelevant, such that starting point effects dissipate for long reaction times. However, if the identity of the stimuli is still uncertain even after a long evaluation (as can happen in conditions with strong across-trial difficulty variability), observers may not be able to resolve the correct option even after long deliberation. In this scenario a decision maker would still want to go for the biased choice by building bias into their slow responses, which would load onto the drift criterion parameter. Although our data is in line with this interpretation, more research would be needed to fully grasp the effect of across-trial variability on drift criterion and starting point.

In line with White and Poldrack (2014), we show that sensory manipulations load more strongly on drift criterion, while non-sensory manipulations load preferentially on starting point. However, we also show that the payoff and base rate manipulation do not load exclusively on either starting point or drift criterion, meaning that drift criterion biases should not be necessarily interpreted as biases in perception. Instead we interpret the different drift diffusion parameter profiles resulting from different bias manipulations as providing converging evidence that perceptual and non-perceptual biases (as identified with our controlled reproduction task) also have different underlying psychological bases.

### Bias manipulations in consciousness research

Reward schemes and prior expectation manipulations are sometimes used in consciousness research (Meijs et al., 2018, 2019; Pinto et al., 2015; A. White et al., 2022; Wyart et al., 2012). Furthermore, in experiments where ‘seen’ and ‘not-seen’ trials (as reported by observers) are compared against each other, the effects of uncontrolled criterion shifts on (un)conscious perception are often unclear, because there is no way of assessing whether they reflect changes in perceptual or post-perceptual processes (Stein et al., 2016). Our results posit a potential problem for such studies if we consider that some manipulations and/or uncontrolled criterion shifts may affect observers’ responses, but not their subjective experience of the stimuli. The controlled reproduction task we introduce here provides a principled method for assessing whether such criterion shifts reflect changes in conscious experience or not. One important difference between our approach and those looking into conscious perception is that we used a discrimination rather than a detection paradigm. Differences between discrimination and detection setups are manifold. For example, the amount of sensory input in present versus absent trials is asymmetric compared to discrimination paradigms, where stimuli are clearly visible on every trial. Here we clearly show that expectations and rewards in discrimination do not affect conscious experience, but rather have a post-perceptual effect on decision making. Future research may use the reproduction measure we introduce here to tackle the related problem of whether expectation and reward manipulation do affect conscious experience in the context of detecting, rather than discriminating stimuli.

## Conclusion

Decision bias contamination is a prevalent issue in the study of conscious experience. When splitting trials between ‘seen’ and ‘not-seen’, as is often done in the study of conscious perception, the possibility of criterion bias is ever-present, as observers may have different decision criteria driven by perceptual or non-perceptual factors. These issues have been discussed at length before (Duscherer & Holender, 2005; Merikle & Reingold, 1998; Peters et al., 2016; Press et al., 2020; Rahnev & Denison, 2018; Reingold & Merikle, 1990; Schmidt, 2015) and most researchers try to control for this possibility (Iemi & Busch, 2018; Peters & Lau, 2015). Here we introduced a novel controlled reproduction method that for the first time allows one to distinguish between decisional and perceptual biases without relying on reverse inference from neural data or physiological data, or on the implied nature of a manipulation. While offering a straightforward method to identify manipulations that affect conscious perception, the results of our study also highlight the importance of further assessing whether explicit bias manipulations or uncontrolled criterion shifts in consciousness research do indeed reflect shifts in conscious perception as claimed before.

## Material and Methods

### Participants

All experimental procedures were approved by the University of Amsterdam Ethics Review Board. Electronic or in paper informed consent was obtained in accordance with the approved procedures. In all experiments, participants were students from the University of Amsterdam recruited through the university lab pool website. After filtering, 138 participants (mean age 20.1, 91 females) completed the *Bias and length reproduction* experiment online and 220 participants (mean age 21, 286 females) completed the *Computational modeling* experiments (50 participants in experiment 1, 86 participants in experiment 2, and 84 in experiment 3). All experiments lasted roughly an hour. Participants were rewarded with 10 euros or 1 research credit per hour, and they could earn up to 5 euros or 0.5 research credits extra based on their number of mistakes during the experiment. On average participants received the same extra reward across conditions and experiments. We removed participants that failed to perform above chance in the discrimination task (SDT *d’* <= 0) and participants whose staircase, SDT criterion, SDT *d’*, and reproduction error fell outside four standard deviations from the sample mean, this is, orthogonal to the conditions of interest. In total 12 participants were removed in the *Bias and length reproduction experiment (Müller-Lyer: three participants, base rate: two participants, payoff: seven participants)* and 13 in the *Computational modeling* experiments (Müller-Lyer: two participants in experiment 1 and 2, and five participants in experiment 3; payoff: two participants in experiment 2; base rate: two participants in experiment 3). In the *Bias and length reproduction* experiment we collected the data of 30 participants on each of the three bias manipulation conditions, removed outliers, and then ran a Bayesian t-test between the biased to long and short conditions in the decision and in the reproduction task. If there was moderate evidence for the effect of our manipulation in both tasks (for either the null or the alternative hypothesis), we stopped data collection (BF^10^ > 3 or BF^10^ < 0.3), otherwise we collected five more subjects and repeated the process. In the *Computation modeling* experiments we aimed to collect 50 subjects in all conditions. However, this was not possible in some conditions (see the Design section below for more details).

### Stimuli

The *Bias and length reproduction* experiment was scripted using Javascript and PsychoJS, and run online through Pavlovia, while the *Computational modeling* experiment was scripted and ran in university behavioral laboratories using Psychopy (Peirce et al., 2019) and Python (Rossum et al., 2010). In all experiments the reference line was 350 pixels long. In the online experiment the monitor resolution varied as each participant completed the experiment on their own computers due to the restrictions imposed by the COVID-19 pandemic in the Netherlands. For the data collected in the lab, stimuli were presented on a 23” (58.4 cm) monitor with a resolution of 1920x1080, at a distance of approximately 75 cm. The size of each pixel is 0.265 mm, or 0.02 visual angle degrees at 75 cm. Depending on the bias source condition, target lines were presented either flanked by inward- or outward-pointing arrowheads (subtending a 45- or 135-degree angle), or by perpendicular lines. In all datasets (except for experiment 1 of the *Computational modeling* experiments) the target line was randomly shifted off-center horizontally (5-9 pixels) to prevent participants from using the endpoints instead of the entire target length and/or using landmarks on or around the monitor to estimate line length.

### Staircase procedure and target line distribution

For both the online and lab collected data, the difficulty of the experiment (length difference between the target line and reference line length) was titrated for each participant by using a staircase procedure that aimed to identify the Just Noticeable Difference point (75% hit-rate) between chance and perfect performance discriminating the length of the target and the reference line. The staircase started at 20 pixels and was updated on a trial-by-trial basis using the weighted up-down method as described by Kaernbach (1991). In all experiments observers completed 25 reversals but only the last 20 reversals were used to calculate the final threshold value (except in experiment 1 of the *Computational modeling* experiments where observers completed 22 reversals and all of them were used to calculate the final threshold). The distribution of target lines consisted in two normal five-value distributions centered on the length of the reference line plus or minus the staircase threshold, depending on the identity of the target line (shorter or longer than the reference line).

### Bias and length reproduction experiment

#### Design

We used three between-subjects bias sources (Müller-Lyer/base rate/payoff) and two within-subjects bias directions (short/long) (see Fig. 1B).

#### Tasks and trial layout

Participants had to categorize target lines as shorter or longer than a reference line (*length categorization task*) or had to reproduce the length of the target lines presented (*length reproduction task*). The experiment was divided into mini-blocks that consisted of five trials. Each mini-block started with the presentation of the reference line followed by a categorization or reproduction trial. Each trial started with a 500 ms fixation period, followed by the target line (500 ms), followed by a second fixation period of variable duration (600, 700 or 800 ms), and ended with the prompt to indicate which one of the two tasks the participant had to complete (length categorization or length reproduction). Crucially, this meant that participants did not know which task they had to perform until after stimulus offset.

#### General procedure

For each task, participants received extensive instructions and extensive practice (all presentation code can be found at the supplementary repository). For the line length categorization, participants first completed 10 trials with feedback with no performance demands, then had to complete 10 correct practice trials in a row with feedback, 10 correct practice trials without feedback and finally, a longer, more difficult block of 25 trials without feedback with at least 80% correct responses. Then, participants completed a staircase procedure to determine the difficulty (length difference between the reference and target line) that yielded a 75% hit rate. Participants then received instructions for the reproduction tasks in the same way it was described for the length categorization task. Finally, participants completed 25 practice trials where both tasks (categorization and reproduction) were intermixed, just as in the actual experiment (see Supplementary fig. S12 for a graphical depiction of the procedure). After the tasks’ instructions and practice, participants in the payoff and base rate conditions were instructed about the asymmetrical punishment and stim-prevalence scheme just before the experimental trials started. For each manipulation we checked whether they understood the base rate and payoff contingencies by showing them examples of the payoff/base rate scheme and asking them which option would maximize their reward if they were unsure about their answer. Participants were required to correctly identify the option that maximized their reward 10 times in a row before continuing. In the Müller-Lyer condition participants were explicitly instructed to ignore the flanking arrowheads and to solely judge the length of the horizontal target lines. In the payoff and base rate condition a similar instruction was given about the flanking vertical lines. The experiment was divided into two 300 trial blocks, one for each bias direction (meaning that the arrow directions, the cost for each incorrect responses and the prevalence of short and long lines did not change within each 300 trial block), of which there were 150 trials per task (categorization/reproduction), summing up to 600 trials. Each participant was assigned to randomly start either with the biased-to-long or biased-to-short condition. During the experiment, participants had a self-paced break after every 50 trials, during which they received block-level feedback on the number of categorization and reproduction mistakes. In the payoff and base rate condition the number of wrong categorization answers was detailed by indicating how often they incorrectly answered ‘short’ or ‘long’, while in the Müller-Lyer condition they were informed about the overall number of incorrect responses. In all conditions the reproduction feedback consisted of the overall number of reproduction errors, defined as a deviation of more than 40 pixels from the actual length of the reference line, regardless of the direction of the error.

### Computational modeling experiments

#### Design

In experiment 1, we employed a full within-subject, two bias source (Müller-Lyer and payoff) by two bias direction (short/long) design. In experiment 2 we used a two between-subjects bias source (Müller-Lyer and payoff) by two within-subjects bias direction design (short/long). Additionally, within the Müller-Lyer condition there were four different arrowhead lengths (30, 40, 50 and 60 pixels long). Finally, experiment 3 consisted of two parts, one full-within, two bias source (Müller-Lyer and payoff) by two bias direction (short/long) design, as described for experiment 1, plus a group of participants that completed two bias directions (short/long) in the base rate condition only. In all three experiments the payoff deduction values and base rate proportion were the same as in the bias and length reproduction experiment, except for experiment 1 where the deduction in the payoff condition was -2/-4. Due to the restrictions imposed by the COVID-19 pandemic in the Netherlands, a group of participants in the base rate condition completed the experiment at the lab while a second group did it online.

#### Tasks and trial layout

In all experiments participants were presented with a reference line followed by five target lines they have to categorize as shorter or longer than the reference. Before the target lines there was a fixation period of 500 ms, except for experiment 1 where it was 700 ms Additionally, in experiments 1 and 3 participants were also asked to estimate the average length of the last five target lines seen (the data of the average length estimation task is not analyzed in this paper).

#### General procedure

The instruction, practice and staircase procedure was as described for the *Bias and length reproduction experiment*, with the following exceptions. During the discrimination instructions and staircase sections of experiment 1 the target lines were flanked by vertical additions. During the categorization instructions participants only completed 10 trials with feedback and 10 trials without feedback. In the payoff and base rate instructions of experiment 1 participants were given examples of payoff and base rate contingencies but were not asked which option would result in maximizing their final reward (see Supplementary fig. S9 for a graphical depiction of the procedure). In experiment 1 the number of trials per task and condition was the same as in the *Bias and length reproduction experiment*, and the same applied for the Müller-Lyer and Payoff condition of experiment 3. In experiment 2, in the Müller-Lyer condition, participants completed 150 trials per task, arrowhead length (4) and bias direction (2), summing up to 1200 trials. In the payoff condition of experiment 2 and in the base rate condition of experiment 3, participants completed 150 trials per task and bias direction combination, summing up to 600 trials. In all three experiments the bias direction within each bias source was blocked so the direction of the arrowheads, cost for incorrect responses, and proportion of short and long lines was constant within each block. As before described for the *Bias and length reproduction experiment*, participants received block-level feedback on the number of mistakes made every 50 trials. Additionally, in the base rate condition the feedback only indicated the total number of correct and mistakes in the decision and reproduction task, regardless of the direction of the error.

#### Analysis

All analysis scripts can be found at https://osf.io/pfe46/?

view_only=7fd0ae3211da433191dfec02ea5edf39

#### Bayesian model comparison

We adopted the Bayesian model comparison framework to test for ordinal-constrained models. This framework allows one to turn relations that are articulated verbally into models of ordinal relations (e.g. condition A > B etc). These statistical models can then be compared using Bayes factor model comparison (see Rouder et al., 2018 for an introduction). The ordinal-constraint approach is described in Haaf et al. (2018) and is based on Klugkist et al. (2005), encompassing prior approach. We started with an unconstrained model (model A) that consisted in all three manipulations having an effect (bias-to-long > bias-to-short) and from there we devised alternative models where one or more conditions didn’t have an effect (models B through G). To further explore the ordinal relationship between the bias manipulations, we constructed a second set of models that further constrained the ordinal relationship between the size of the effects of the manipulations used (e.g. the effect size of the Müller-Lyer condition is smaller than the base rate and payoff effect; models AA through MM). For the unconstrained model, we use a g-prior approach as described in Rouder et al. (2012) with a default setting on the scale of effect, r = √2/2. The other models are restricted versions of the unconstrained model using ordinal and equality constraints. For the analysis, we used the BayesFactor package in R (Morey & Rouder, 2018). For a graphical depiction of all the models see Supplementary Figure S3.

#### Signal detection analysis

To determine performance and bias on the tasks we computed signal detection sensitivity (*d’*) and criterion (*c*) based on hit rate and false alarms as follows:

$$d^{\prime }=Z(HR)-Z(FAR)\;\;\text{and}\;\;c=\frac{1}{2}\times (Z(HR)+Z(FAR))$$

Where *Z()* denotes the inverse of the standard normal cumulative distribution (often denoted as the Z-transform, as it has a mean of 0 and a standard deviation of 1). The formula can be easily translated to R code by replacing the *Z()* with the *qnorm()* function from the R stats package. HR denotes hit rate, FAR denotes false alarm rate. In this setting correct ‘long’ responses are considered hits and correct ‘short’ responses correct rejections.

#### Drift diffusion models

In the *Computational modeling experiments* we fitted a series of drift diffusion models (Ratcliff & McKoon, 2008) to the RT distributions of ‘long’ and ‘short’ responses. To fit the model we use the hierarchical Bayesian implementation of the HDDM toolbox (Wiecki et al., 2013) (version 0.8.0). Fitting the model to ‘long’ and ‘short’ responses (usually termed as ‘stimulus coding’) allowed us to estimate parameters that could have induced biases in participants’ behavior. The full posterior distributions of the estimated parameters are generated by a Bayesian MCMC and allow us to quantify not only the most likely parameter value but also the uncertainty associated with that estimate. We ran three separate Markov chains Monte Carlo with 30.000 samples each. Of those, 9.000 were discarded as burn-in, and we applied a thinning factor of 2. Individual parameter estimations were then obtained from the remaining 10.500 samples. All group-level chains were visually inspected to ensure convergence. We also computed the Gelman-Rubin Ȓ statistic to compared within and between chain variance, and checked that all group level estimates had an Ȓ between 0.99 and 1.01. To account for contaminants we filter all trials with reaction times faster than 200 ms and slower than 4 SD over the mean of each experiment sample.

## Supplementary Material

Supplementary Materials

## Box 1 Significance statement

Our decisions are shaped both by stimuli in our environment and the expectations we have about the world. For example, you may be inclined to say you perceived something if you are rewarded for detecting it. Nevertheless, it is unknown if such non-sensory information actually affects subjective experience, or whether it merely changes the likelihood of giving a certain response. Here, we answer this question by developing a novel task that directly asks people to reproduce their conscious experience. Using this task we show that subjective experience is affected by visual illusions, but not rewards or expectations. Additionally, we show that the modeling of response times, although informative, is insufficient to distinguish whether information affects decisions or also sensory experience.
