# Supplementary material for: Predictions and rewards affect decision-making but not subjective experience
Source: Proc Natl Acad Sci U S A. Author manuscript; Available in PMC 2024 Mar 18. (PMC10622870; doi:10.1073/pnas.2220749120)
Supplement: Supplementary Materials [file EMS194652-supplement-Supplementary_Materials.pdf]

Supplementary materials and methods

S1: Sensitivity in the length categorization task

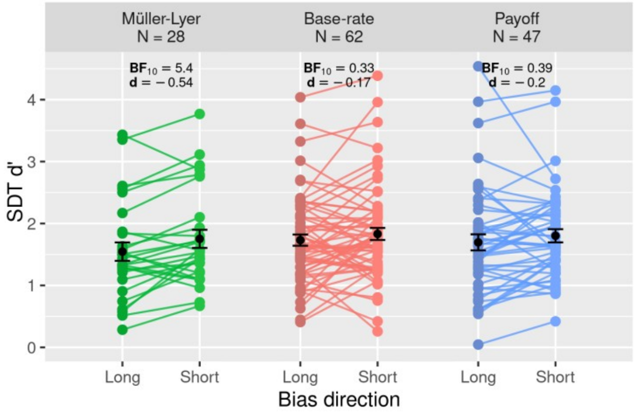

Supplementary Figure S1: Categorization sensitivity. SDT d' values for each subject are plotted along with the group average for each bias source and bias direction condition. All error bars are standard errors of the mean. Higher values indicate a better performance at the task. BF values correspond to a Bayesian t-test with a default Cauchy prior of $\sqrt{2} \div 2$. *d* values correspond to Cohen's d effect size coefficients.

S2: Length reproduction and target line length correlation

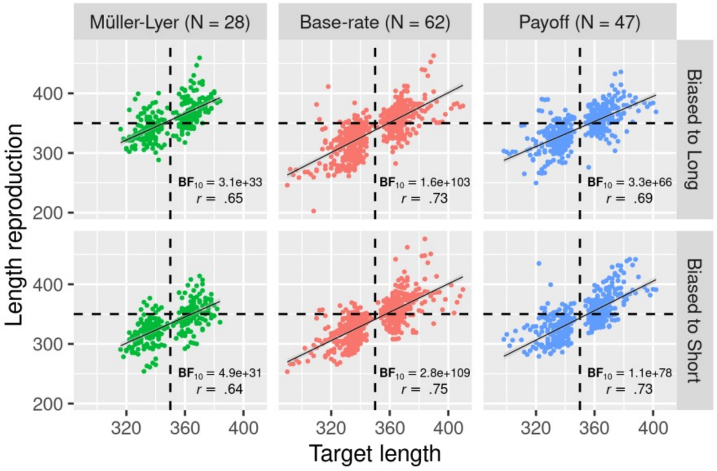

Supplementary Figure S2: Length reproduction and target length correlation. Correlation between length reproductions (y-axis) and target line length (x-axis) for each bias manipulation separately for each bias direction. Each point represents the average length reproduction of a subject on a given bias source and bias direction condition for a given target length. The correlation was then calculated on the pooled data of all subjects. The correlation test corresponds to a Bayesian test for a true linear correlation (*rho*) between length reproductions and target line lengths with a 'medium' prior (⅓). The *rho* coefficient is the mean value of a MCMC posterior sample consisting of 10,000 iterations.

S3: Bayesian ordinal models

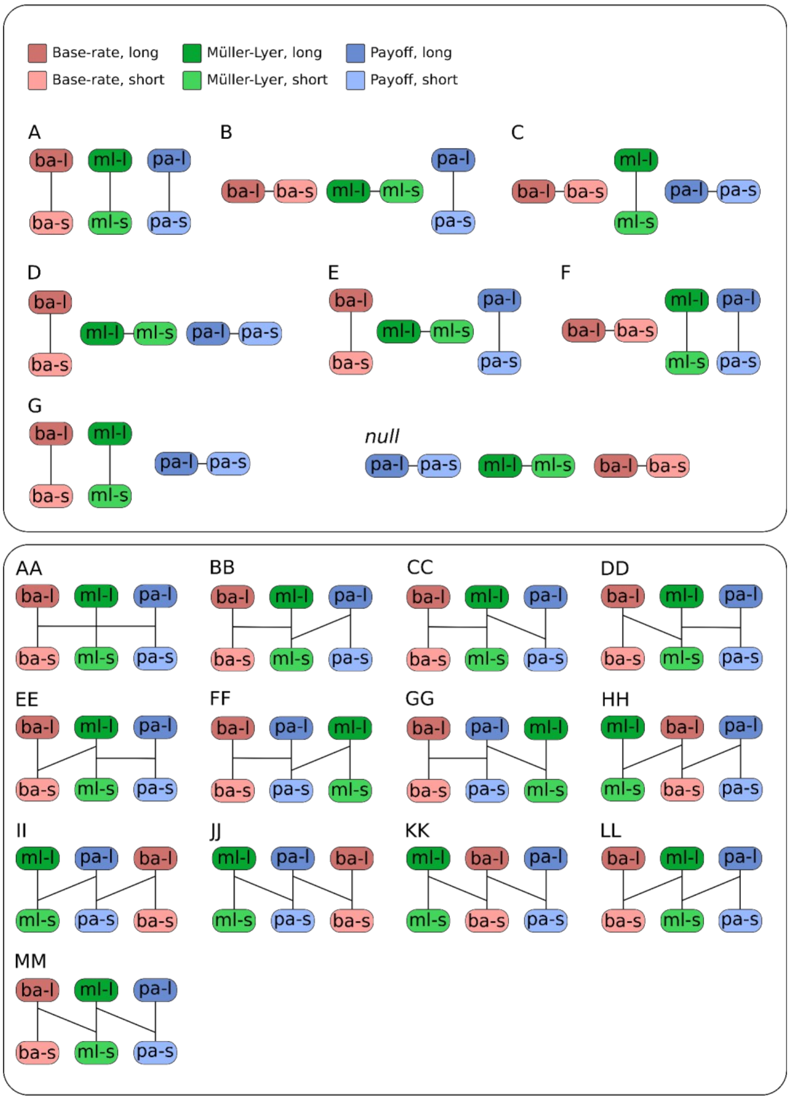

Supplementary Figure S3: Bayesian ordinal models. Each cell refers to the mean SDT criterion, reproduction error or DDM trace value depending on the section of the results where the models cited. On each cell ‘ba’ refers to base rate, ‘ml’ to Müller-Lyer and ‘pa’ to payoff. While ‘l’ refers to biased to long and ‘s’ biased to short. Vertical lines connecting cells indicate that the top cell has a higher value than the bottom cell (non-zero positive effect size), while horizontal lines between cells indicate null-effects. For any of the models in the top panel, the relationship between effects within the models are not constrained. As a result, effect sizes within a model do not have to be the same, the models only specify whether the bias manipulations have zero or non-zero effects. The ordinal relationship between effect sizes for different bias source conditions is tested in models in the bottom panel, when effects (vertical lines) are connected by a horizontal line (effect size is the same) or diagonal line (in which case the effect connected to the upper part of the diagonal line is modeled as larger than the effect that is connected to the lower part of the diagonal line). For example, in model AA, the effect sizes of all three bias manipulations are the same size, while in model BB the size of the base rate and the Müller-Lyer conditions is the same, and both are smaller than the payoff effect.

S4: Bayesian ordinal models BFs values - Decision bias and length reproduction experiment

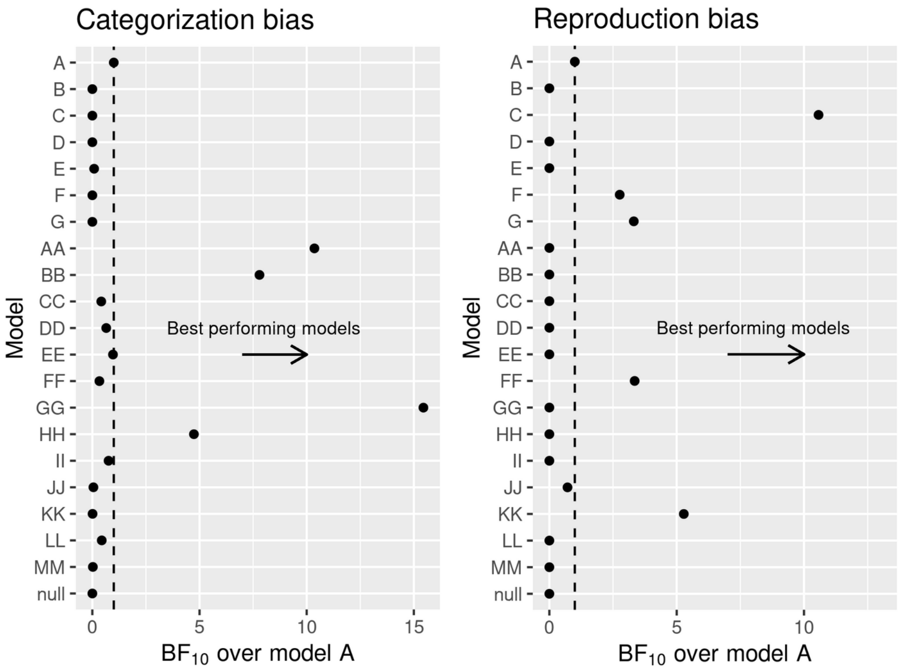

Supplementary Figure S4: Ordinal model comparison. The Bayes factor of each model cannot be directly compared unless they share the same denominator. Therefore, to establish which model performs the best, we first compare the Bayes factor of each model against model A (for example, the Bayes factor of model GG reported in the results section corresponds to the Bayes factor of model GG over the Bayes factor of model A, or model GG BF^{10} / model A BF^{10}). The BF value (x-axis) for each model indicates how much more likely that model is over model A. The vertical dashed line separates models that perform worse than model A (left side) from the ones that overperform it (right side). The best performing models are model GG (categorization task) and model C (reproduction task).

S5: Decision bias and reproduction error by block

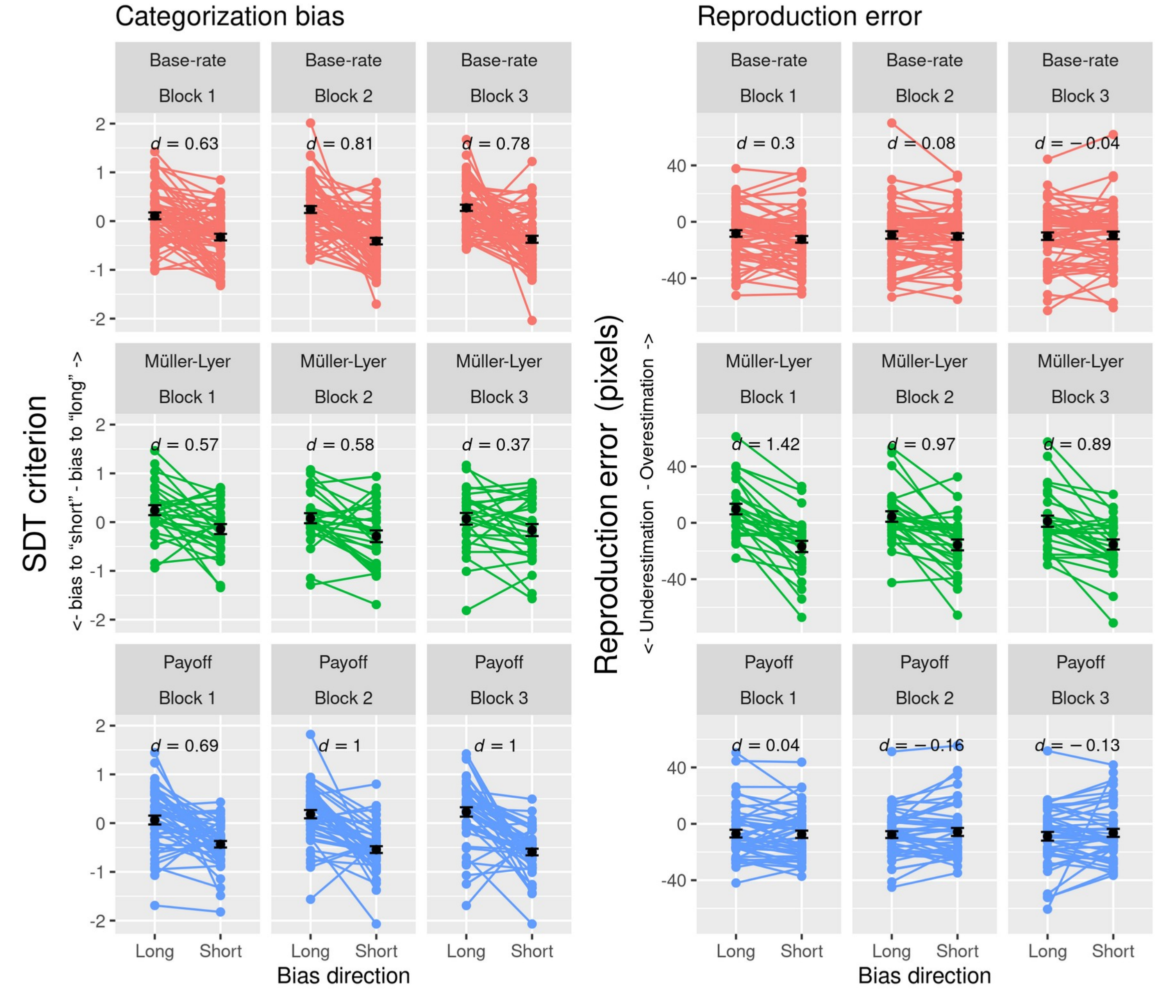

Supplementary Figure S5: Categorization bias and reproduction error by experimental block. A) Categorization bias. The SDT criterion value for each subject along with the group average for each bias source and bias direction. Higher values indicate a stronger bias towards answering 'long' while lower values indicate a stronger bias towards answering 'short'. **B) Reproduction error.** The average reproduction error (length reproduction - target length) for each subject is displayed for each bias source and bias direction condition. Higher values indicate lines are reproduced as longer than the target line while lower values indicate lines are reproduced as shorter than the target line. **A)** and **B)** The experiment was divided into three blocks of 100 trials (50 trials per task) and then categorization bias and reproduction error was calculated for each block. All error bars indicate the standard error of the mean. To test for an effect of time (experimental block) we fit separately for each bias manipulation and task two models where either bias direction or the interaction of bias direction and block was used as independent variables. In the decision task the model with only bias direction better explained the data across all bias manipulations ($BF_{main\text{-}over\text{-}interaction}$ = 62, 18 and 23, respectively for the base rate, Muller-Lyer and payoff condition). As before in the reproduction task the model with only bias also better explained the data across all bias manipulations ($BF_{main\text{-}over\text{-}interaction}$ = 78, 6 and 193, respectively for the base rate, Muller-Lyer and payoff condition).

S6: Drift diffusion modeling fitting predicted data

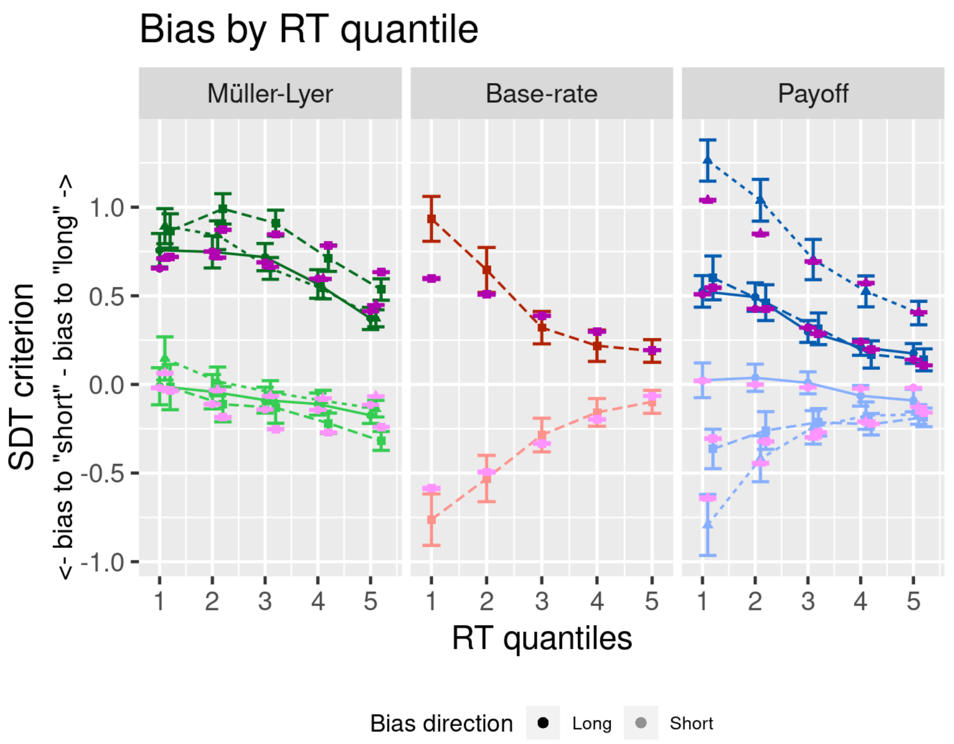

Supplementary Figure S6: DDM model fitting: empirical and predicted data. The same data as in Fig. 3B. Using the fitted parameter values of the DDM model, simulated data was generated (500 iterations). As with the empirical data, we binned the reaction times of each bias manipulation into quintiles (x-axis) and calculated decision bias (SDT criterion; y-axis). Empirical data is plotted in green, red or blue depending on the bias condition, and simulated data is plotted in purple for the long bias direction, and pink for the short bias direction. All error bars show the standard error of the mean.

S7: Bias and sensitivity computational modeling experiments

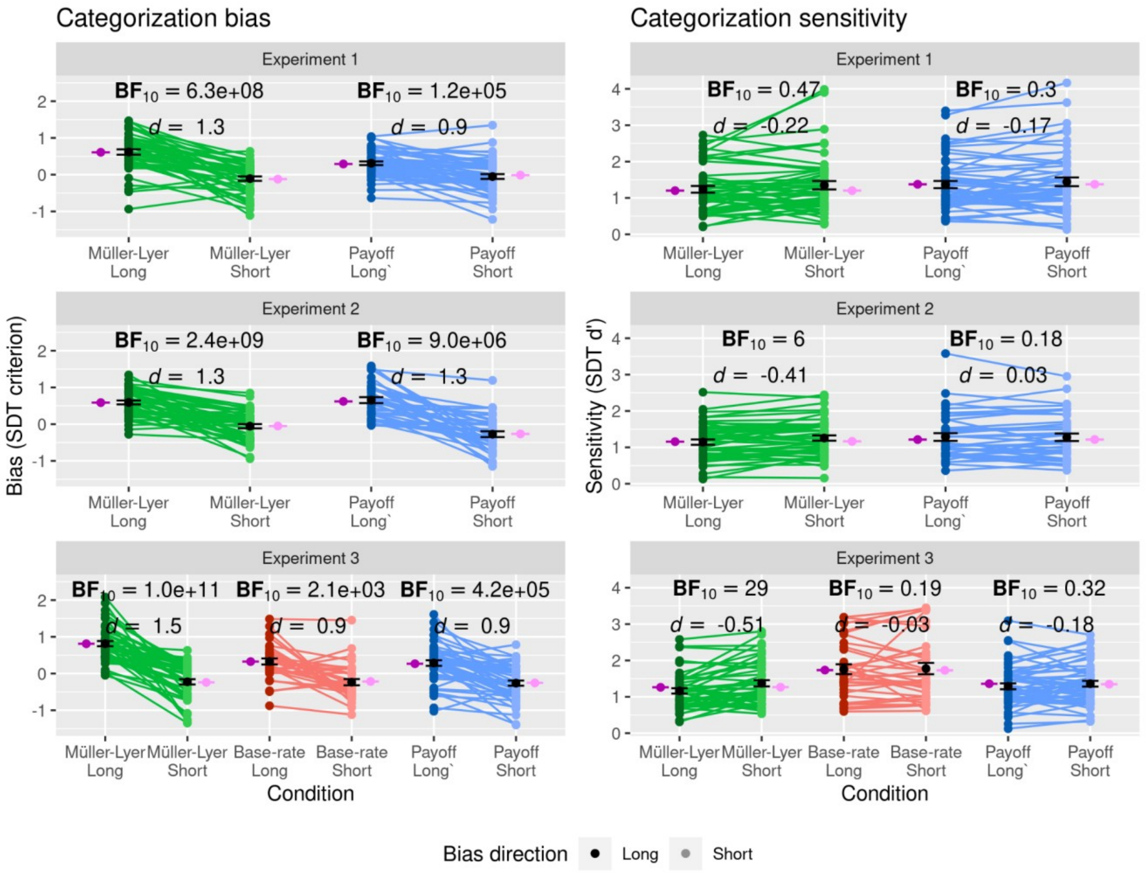

Supplementary Figure S7: Categorization bias and sensitivity computational modeling experiments. The SDT criterion (left column) and d' (right column) values for each subject are displayed along with the group average for each bias source and bias direction condition. Empirical data is plotted in green, red or blue depending on the bias condition, and data simulated using the fitted parameter values of the DDM analysis is plotted in purple for the long bias direction, and pink for the short bias direction. All error bars show the standard error of the mean. On bias plots (left column) higher values indicate a stronger bias towards the 'long' choice and lower values towards the 'short' choice, on the sensitivity plots (right column) higher values indicate better performance at categorizing short and long lines. BF values correspond to a Bayesian t-test with a default Cauchy prior of $\sqrt{2} \div 2$. *d* values correspond to Cohen's d effect size coefficients.

S8: Reaction time quantile probability plot

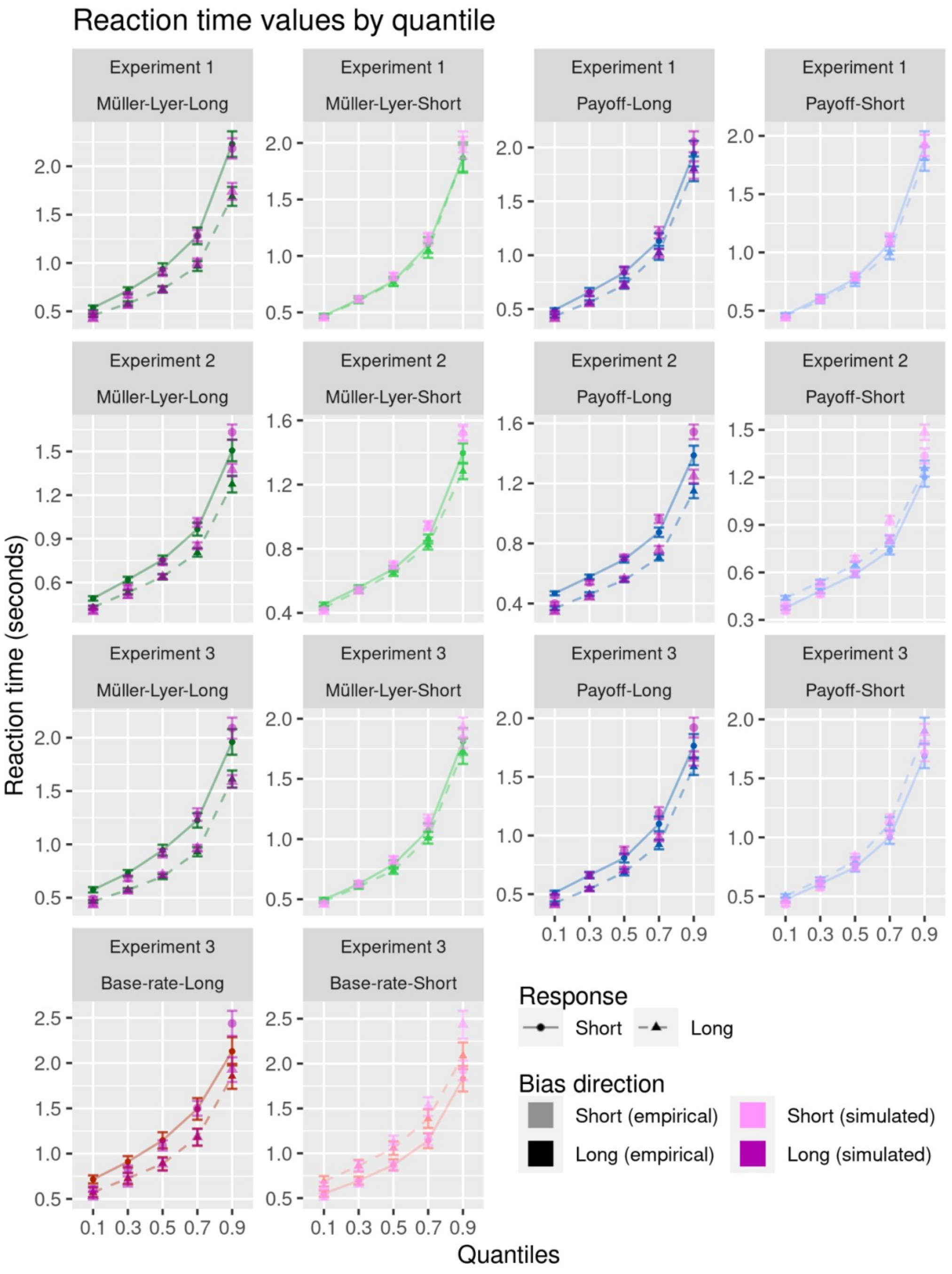

Supplementary Figure S8: DDM model fitting: empirical and predicted reaction time quantile probability plot. Using the fitted parameter values of the DDM model, simulated data was generated (500 iterations). As with the empirical data, for each participant we binned the reaction times of each bias manipulation into quintiles (x-axis) and plotted the simulated data over the empirical data. Empirical data is plotted in green, red or blue depending on the bias condition, and simulated data is plotted in purple for the long bias direction, and pink for the short bias direction. All error bars show the standard error of the mean.

S9: Drift diffusion model parameters full posterior distribution

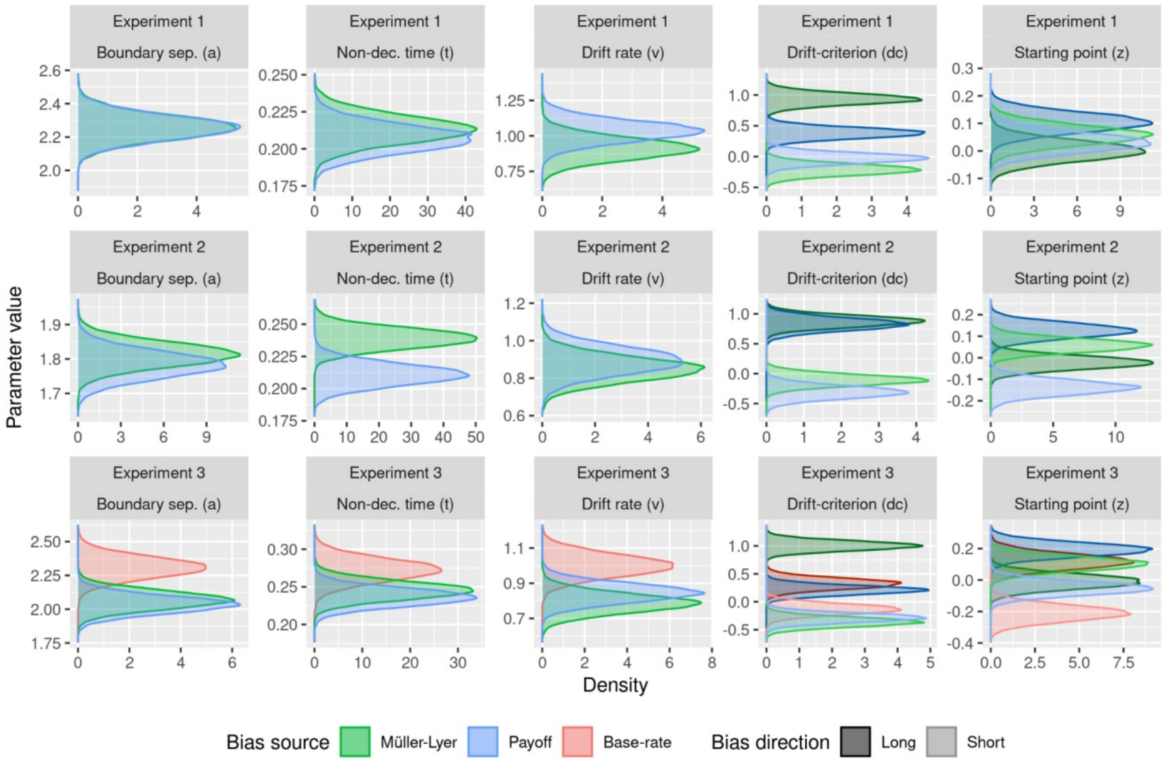

Supplementary Figure S9: Group-level posterior distributions of all DDM parameters. The boundary separation, non-decision time and drift rate parameters were fixed within bias sources, so one estimation was made for both biased to short and long conditions within each bias manipulation. Drift criterion and starting point were allowed to vary within bias sources so each bias direction was estimated separately. The drift criterion and starting point distributions are plotted as single point estimates in Figure 3C.

S10: Drift diffusion modeling fitting predicted data

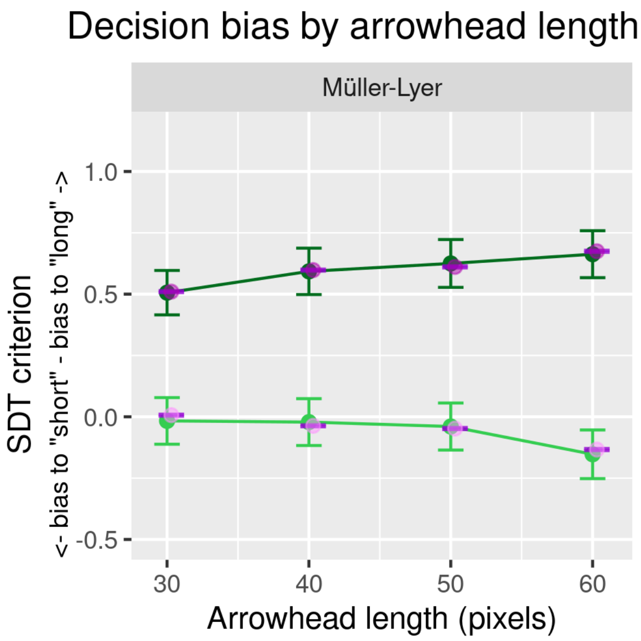

Supplementary Figure S10: DDM model fitting: empirical and predicted data. Using the fitted parameter values of the DDM model simulated data was generated (500 iterations). As with the empirical data, we calculated the SDT criterion for each arrowhead length and for each bias direction. Higher values indicate a stronger bias towards answering 'long' while lower values indicate a stronger bias towards answering 'short'. Empirical data is plotted in green, and simulated data is plotted in purple for bias direction long, and pink for bias direction short.

S11: Reaction time quantile probability plot

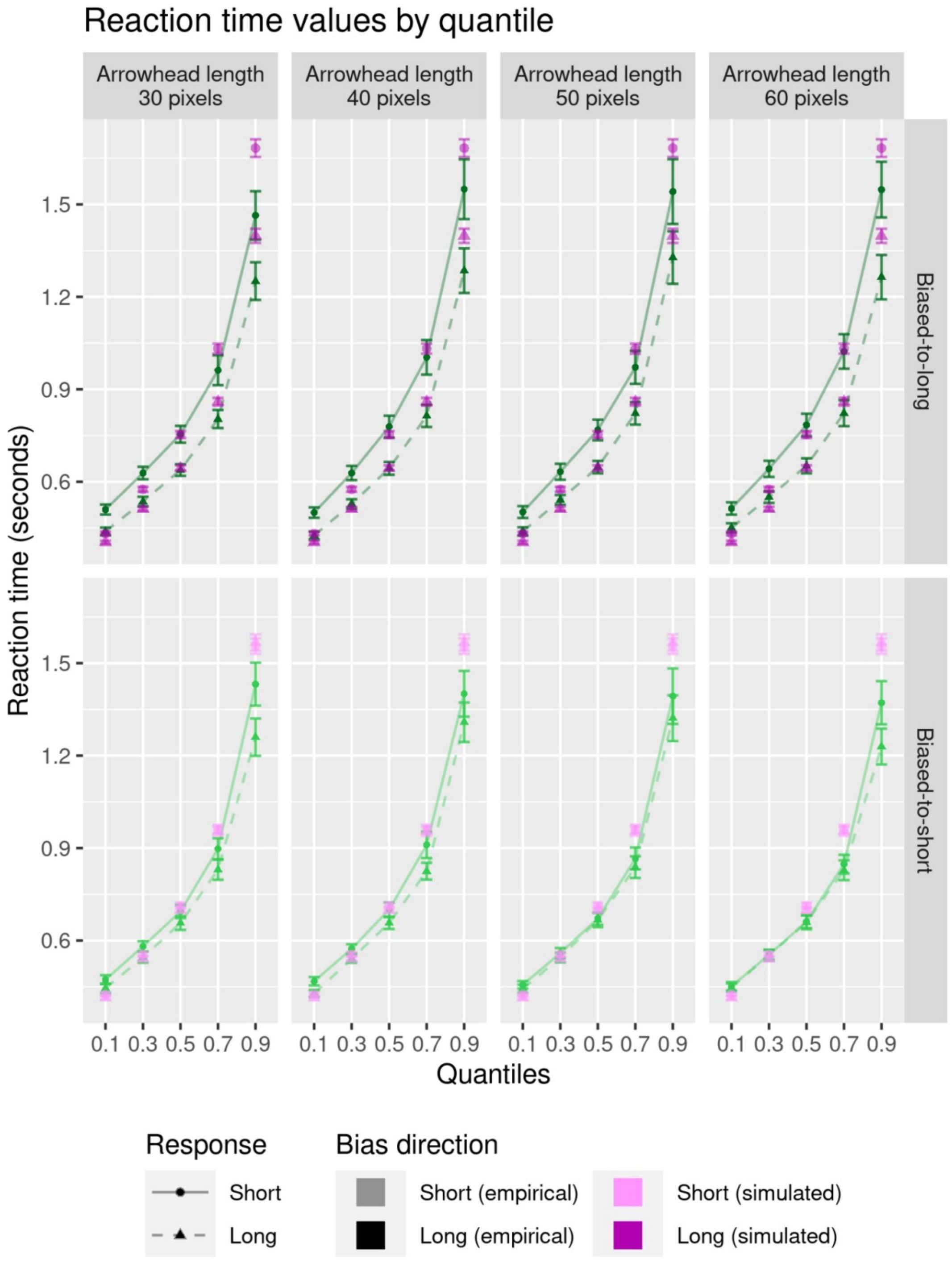

Supplementary Figure S11: DDM model fitting: empirical and predicted data arrowhead length data. Using the fitted parameter values of the DDM model, simulated data was generated (500 iterations). As with the empirical data, for each participant we binned the reaction times of each bias manipulation into quintiles (x-axis) and plotted the simulated data over the empirical data. Empirical data is plotted in green, red or blue depending on the bias condition, and simulated data is plotted in purple for the long bias direction, and pink for the short bias direction. All error bars show the standard error of the mean.

S12: Experiment general procedure

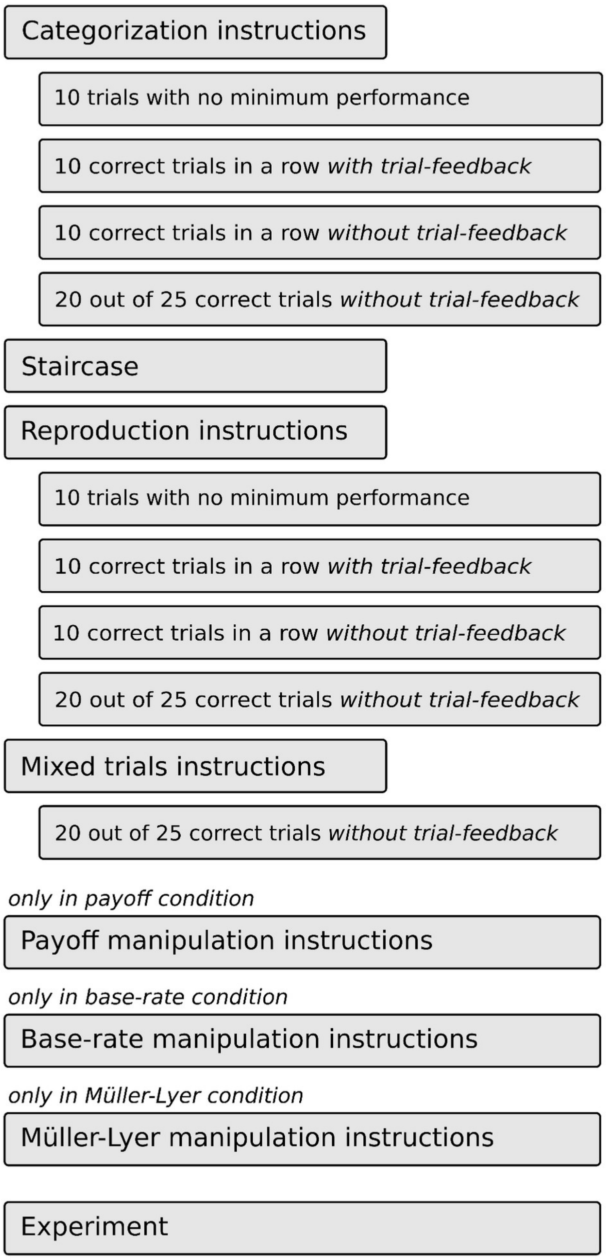

Supplementary Figure S12: Experiment general procedure of both the bias and length reproduction experiment, and computational modeling experiments. For each task participants received extensive instructions and completed multiple practice trials. All participants completed the categorization instructions, staircase, reproduction instructions and mixed trials instructions. Depending on the bias manipulation condition participants received specific instructions related to the manipulation. From top to bottom the Figure depicts the order and stages of each part of the instructions. When there was a minimum performance requirement in any of the practice trials sections, participants repeated the practice block until they achieved the expected performance. In the computational modeling experiments participants completed the categorization instructions, staircase and payoff, base rate and Muller-Lyer instructions with some minor changes. See General procedure in Methods and Materials for more details.
